# Frequency of the Dopamine Receptor D3 (rs6280) vs. Opioid Receptor µ1 (rs1799971) Polymorphic Risk Alleles in Patients with Opioid Use Disorder: A Preponderance of Dopaminergic Mechanisms?

**DOI:** 10.3390/biomedicines10040870

**Published:** 2022-04-07

**Authors:** Marjorie C. Gondré-Lewis, Igor Elman, Tanya Alim, Edwin Chapman, Beverlyn Settles-Reaves, Carine Galvao, Mark S. Gold, David Baron, Shan Kazmi, Eliot Gardner, Ashim Gupta, Catherine Dennen, Kenneth Blum

**Affiliations:** 1 Neuropsychopharmacology Laboratory, Department of Anatomy, Howard University College of Medicine, Washington, DC 20059, USAcarineggalvao@gmail.com (C.G.); 2 Department of Psychiatry, Cambridge Health Alliance/Harvard Medical School, Cambridge, MA 02139, USA or dr.igorelman@gmail.com; 3 Department of Psychiatry and Behavioral Sciences, Howard University College of Medicine, Washington, DC 20059, USA; talim@howard.edu (T.A.); echap1647@aol.com (E.C.); 4 Department of Psychiatry, Washington University School of Medicine, St. Louis, MO 63110, USA; drmarkgold@gmail.com; 5 Graduate College, Western University Health Sciences, Pomona, CA 91766, USA; dbaron@westernu.edu; 6 College of Osteopathic Medicine of the Pacific, Western University of Health Sciences, Pomona, CA 91766, USA or shan.kazmi@western.edu; 7 Neuropsychopharmacology Section, Intramural Research Program, National Institute on Drug Abuse, National Institutes of Health, Baltimore, MD 21224, USA; egardner@mail.nih.gov; 8 Future Biologics, Lawrenceville, GA 30043, USA; ashim6786@gmail.com; 9 The Kenneth Blum Behavioral & Neurogenetic Institute, Austin, TX 78701, USA; catherine.a.dennen@gmail.com; 10 Department of Clinical Psychology and Addiction, Institute of Psychology, Faculty of Education and Psychology, ELTE Eötvös Loránd University, Egyetem tér 1-3, 1053 Budapest, Hungary; 11 Department of Psychiatry, School of Medicine, University of Vermont, Burlington, VT 05405, USA; 12 Centre for Genomics and Applied Gene Technology, Institute of Integrative Omics and Applied Biotechnology, Nonakuri, Purba Medinipur 721172, West Bengal, India; 13 Department of Psychiatry, Wright State University Boonshoft School of Medicine and Dayton VA Medical Centre, Dayton, OH 45324, USA

**Keywords:** opioids, GARS, dopaminergic mechanisms, DRD3, OPRM1, mesolimbic circuitry, brain reward cascade, African American, opioid epidemic, epigenetics, genetic variations

## Abstract

While opioids are a powerful class of drugs that inhibit transmission of pain signals, their use is tarnished by the current epidemic of opioid use disorder (OUD) and overdose deaths. Notwithstanding published reports, there remain gaps in our knowledge of opioid receptor mechanisms and their role in opioid seeking behavior. Thus, novel insights into molecular, neurogenetic and neuropharmacological bases of OUD are needed. We propose that an addictive endophenotype may not be entirely specific to the drug of choice but rather may be generalizable to altered brain reward circuits impacting net mesocorticolimbic dopamine release. We suggest that genetic or epigenetic alterations across dopaminergic reward systems lead to uncontrollable self-administration of opioids and other drugs. For instance, diminished availability via knockout of dopamine D3 receptor (DRD3) increases vulnerability to opioids. Building upon this concept via the use of a sophisticated polymorphic risk analysis in a human cohort of chronic opioid users, we found evidence for a higher frequency of polymorphic DRD3 risk allele (rs6280) than opioid receptor µ1 (rs1799971). In conclusion, while opioidergic mechanisms are involved in OUD, dopamine-related receptors may have primary influence on opioid-seeking behavior in African Americans. These findings suggest OUD-targeted novel and improved neuropharmacological therapies may require focus on DRD3-mediated regulation of dopaminergic homeostasis.

## 1. Introduction

Worldwide, about 16 million are afflicted with opioid use disorder (OUD), and approximately 100,000 people die of opioid overdose each year [1,2]. The United States (US) statistics are even more alarming, pointing to 101,260 overdose deaths in 2021 [3] that have been shortening life expectancy since 2004 [4,5]. Only about 20% of opioid abusers receive the currently approved medication-assisted treatments (MATs): buprenorphine, methadone or naltrexone. Untreated individuals are prone to increased morbidity, mortality and devastating psychosocial and legal consequences [6]. The overdose deaths have become particularly prominent in Black communities where fatality rates have sharply increased and are actually four times greater in older non-Hispanic Black men compared to other persons in the same 55+ age group [7,8]. Certainly, with these well-known facts and consequent devastation, the entire scientific community is being challenged to find real solutions to this unwanted conundrum—as espoused by Collins and Volkow in their call for *“*all hands on deck” [9].

### 1.1. Brain Reward Function

**Figure 1:**
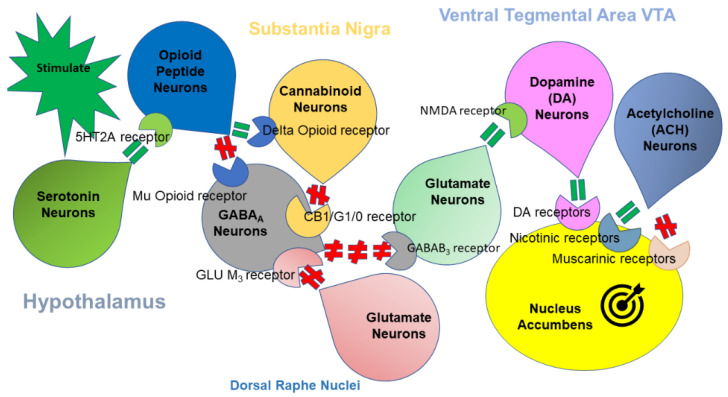
The Brain Reward Cascade with the major neurotransmitter systems involved; green equal sign indicates stimulation and red pound sign stands for inhibition. Stimuli-induced serotonin release in hypothalamus activates 5HT_2a_ receptors leading to release of hypothalamic opioid peptides. The latter exerts opposite effects via two distinct opioid receptors: (a) inhibition via µ opioid receptor, e.g., GABA_A_ neurons in substania nigra and (b) stimulation of cannabinoid neurons (e.g., anandamide and 2-archydonoglcerol) via β–endorphin linked δ receptors inhibiting GABA_A_ neurons at the substania nigra. GABA_A_ neurons in the substania nigra may be also indirectly disinhibited by cannabinoids, 0 in hedonic and motivational responses [20].

Modern scientific exploration has revealed numerous neurotransmitters and second messengers linked to mesocorticolimbic processing of reward and aversion. Dopamine is still attributed the central role [10,11] given its mediation of motivation and learning processes [12,13]. However, mesocorticolimbic dopamine pathways do not function alone; they are rather contained within a multifaceted network of interconnected structures, each of which plays a unique role in pursuit of reward and avoidance of punishment [14,15,16,17]. These cascading interactions generate balanced release of dopamine across numerous nucleus accumbens’ (NAc’) effector regions involved in memory, decision making, salience, pleasure and stress (to name a few). Thus, NAc serves as reward homeostat (also known as hedonostat) sensing dopamine concentrations’ deviations from the set point determined by the neuroanatomical/neurochemical regulators throughout the entire brain [18,19]; these deviations are evoked by the stimuli falling within the entire range of valence from aversive to pleasurable. Figure 1 is a graphic depiction of the Brain Reward Cascade (BRC) comprised of serotonergic, cannabinoidergic, opioidergic, GABAergic, glutaminergic, and dopaminergic (among others) systems defining the overall release of dopamine in the NAc.

### 1.2. Dopaminergic Aspects of Addictive Behvaior

Dopaminergic brain systems play a central role in natural reward and motivation and are the main neural substrates for the actions of abused chemical substances and natural rewards alike [21]. Early discoveries by Blum and Noble of alcoholism-related genes laid the foundation for the modern field of Psychiatric Genetics [22]. Excessive exposure to natural stimuli, like palatable food or high-thrill behaviors (e.g., gaming or gambling) as well as to chemical substances (e.g., opioids or cocaine) may alter brain reward circuits via epigenetic mechanisms [23] and other types of neuroplasticity [17,19,24] such as protracted transcriptional mRNA expression [25] which eventually bring about addictive behavior, characterized by diminished sensitivity to natural rewards (i.e., reward deficiency syndrome (RDS)). RDS can be manifested by compensatory engagement in pursuit of rewarding stimuli regardless of adverse consequences [26,27]. Therefore, RDS has been linked to opioids, other drugs [28,29], and comorbid neuropsychiatric syndromes [30,31,32,33]. These insights supported the definition of “addiction” as a brain disorder [34] by the American Society of Addiction Medicine, spurring cross-fertilizing interactions between clinical and basic research. Nowadays, addiction science is well positioned to comprehend the true nature of the brain disorder through the prism of genomic medicine.

Brain disorders characterized by RDS are diverse and complex from both clinical and genetic standpoints. The difficulty of several genome-wide association studies (GWAS) in finding significant associations with various gene candidates may stem from several factors including clinical heterogeneity, polygenic nature of phenotypic targets, and lack of adequate controls. Such difficulties notwithstanding, using genome wide association studies (GWAS), Hancock et al. [35] identified 11 genetic loci for smoking, 8 loci for alcohol, and 2 loci for illicit drugs combined. Thus, we may need to consider various neurotransmitter-informed RDS subtypes based on their predominantly serotonergic-, cannabinoidergic-, endorphinergic-, opioidergic-, glutaminergic-, or dopaminergic nature. At the same time, it is important not ‘to miss the forest for the trees’ as we are still striving for the discovery of coherent laws that will unite the prevailing models of RDS to generate new leads for the development of therapeutic interventions. DNA polymorphisms, particularly as related to dopaminergic function, is of critical importance for unraveling molecular underpinnings of addiction [36,37] and to address the plausibility of using specific gene editing techniques (e.g., insertion of protective and/or corrective genes) to overcome drug/alcohol seeking behavior in genetically bred drug/alcohol preferring mice [38].

### 1.3. Dopamine D3 Receptor Function and Addiction Vulnerability

The dopamine D_3_ receptor (DRD3) gene is situated on chromosome 3q13.3 and is densely expressed in the limbic subcortical regions, e.g., the NAc, thalamus, hypothalamus and cerebellum [39]. This expression is particularly conspicuous in the basolateral nucleus of the amygdala (BLA), an essential region for processing of opioid-related reward and withdrawal aversion-related memories [40,41] as assessed with conditioned place preference and aversion (in conjunction with molecular analyses of BLA protein expression) [42]. Using genetic manipulation of individual dopamine receptors in animals, our understanding of some molecular and cellular mechanisms inherent in addictive behaviors have improved [42,43,44,45,46,47,48,49,50,51,52,53]. Most of what is known about blocking, activating, gain-of-function studies were through animal studies.

In fact, whereas the intra-BLA DRD3 blockade had no effect in opioid-naive rats, such blockade prevented the establishment of opioid reward and withdrawal aversion memory in the animals that were chronically exposed to opioids [42]. The switch in the functional significance of DRD3 transmission corresponded to significant increases in calcineurin and in Cdk5 phosphorylation, with a proportionate decrease in intra-BLA DRD3 expression. Blocking of either intra-BLA Cdk5 or calcineurin reversed these effects, switching intra-BLA associative memory formation back to a DRD3-independent mechanism. Consequently, DRD3′s single nucleotide polymorphisms (SNPs) have emerged as potential modulators of addiction-related activities in the reward pathway, and their signaling is linked to downstream Cdk5 and calcineurin transmission, both of which are critically involved in memory-related synaptic plasticity [42]. This work supports targeting the DRD3 gene as a therapeutic strategy in addiction (discussed below). Indeed, many studies have revealed that the deletion or functional blockage of the DRD3 receptor is associated with increased vulnerability to heroin or oxycodone intake [30,49,50,51,53], and the DRD3 Ser9Gly SNP is associated with substance abuse and can have an additive effect for negative affective symptoms in Parkinson’s disease [49,50,51,52,53,54]. Other than behavioral outcomes, precise mechanisms for the action of SNPs have not yet been investigated in animal models or humans.

### 1.4. Neurogenetics of Dopamine D3 Receptor in Opioid Use Disorder

As reviewed by Abijo et al. [54], to date, a number of studies have linked DRD3 variants with OUD. The rs2654754, rs9288993, and rs1486009 DRD3 SNPs showed significantly high association with OUD in subjects of predominantly European ancestry. For Han Chinese subjects, the rs6280 and rs9825563 SNPs were significantly associated with the development of early-onset OUD [55]. Prior studies considering DRD3 variants in African Americans or Hispanics have not been identified [54,55,56] (see Table 1). Therefore, the findings in the current study are novel and necessary for considering ethnicity-informed treatments of OUD.

### 1.5. Neurogenetics of the Opioid Receptor µ1 in Opioid Use Disorder

The OPRM1 gene located on chromosome 6q24-q25 encodes the µ opioid receptor, which is a potent mediator of the opioids’ rewarding effects. Several SNPs in OPRM1 have been linked with the misuse of substance like alcohol, cocaine and nicotine. In particular, OPRM1 rs1799971 (A118G) and rs1799972 (C17T) variants are especially associated with OUD [55,66], yet only a handful of studies included subjects of African descent in their research. One study found an association of OUD with rare SNPs, rs199971 and rs17174801 in 1238 people [54]. This same group found an association between rs62638690 (not rs17174794) and opioid and cocaine addiction in European Americans [67]. However, the same SNPs were not analyzed across other ethnic groups for direct comparison. For all ethnicities combined, the difference between opioid dependent vs. nondependent groups was only for minor alleles rs1799972 (T), but not in major alleles’ frequencies. The rs1799971 (G) frequencies in that study was 0.016 African Americans, 0.115 for Caucasians and 0.142 for Hispanics. Thus, African Americans presented the lowest risk allele associated with OUD with rs1799971(G), whereas rs1799971(A) may actually be protective against opioid addiction [68]. In contrast, Hispanic opioid-dependent vs. non-opioid-dependent subjects had higher rs1799971(G) allele frequency. Another OUD study revealed association between polymorphic haplotypes in OPRM1 among Caucasians, but not among African Americans when the following SNPs were considered: -2044C/A, -1793T/A, -1699insT, -1469T/C, -1320A/G, -111C/T, +17C/T (Ala6Val), and +118A/G [69,70]. Likewise, a study by Crowley et al. [71] reported no link between five OPRM1 gene SNPs (e.g., rs1799971 and rs1799972) and OUD in both African Americans and Caucasians, notwithstanding significant allele differences.

On the whole, the reports regarding the OPRM1 and OUD association are inconsistent (Table 2) with both positive [72] and negative [73] outcome studies on the G allele of rs1799971 in Chinese patients. In the Bulgarian population, there was no evidence of association between OUD and rs1799971. Another study [74] even suggested that the G allele of rs1799971 may actually have a protective effect, where at-risk neonates (of unspecified ancestry) with this allele had on average a shorter stay in the hospital and required 25% less neonatal abstinence treatment [74].

Notably, SNP assessments may have therapeutic implications for predicting therapeutic response to methadone treatment [75]. Specifically, SNP alleles A/G and G/G but not A/A of rs10485058 located in the 3′ UTR may be associated with relapse and reduced efficacy of methadone [76]. The G allele mRNA apparently binds to miRNAs, which inhibits translation of MOR, decreasing methadone effect, thus increasing susceptibility to relapse [76].

In short, SNPs associated with OUD in Caucasians may not confer OUD vulnerability in African Americans; some SNPs may be neuroprotective, contributing resilience to OUD. Genetic counseling may prove to have heuristic value for determining the optimal therapeutic effect. Moreover, ethnic ancestry should be taken into consideration for the choice of the optimal MAT agent and in the overall design of the treatment plan.

### 1.6. Genetic Addiction Risk Severity

Geneus Health LLC. scientists, in conjunction with their Genomic Testing Centre (GTC), have successfully developed the first Genetic Addiction Risk Severity (GARS) test to predict liability for addiction and RDS [28,80]. To develop the GARS, ten reward candidate genes were selected, including dopamine receptors (DRD1, 2, 3, 4); Dopamine Transporter (DAT1); serotonin transporter, catechol-O-methyltransferase (COMT), monoamine oxidase (MAO), γ amino butyric acid (GABA), mu opioid receptor 1 (OPRM1) and other SNPs and point mutations that determine the release of dopamine at the brain reward centers. The variants or SNPs, including point mutations, are chosen to reflect a hypodopaminergic trait, based on thousands of association studies providing clear evidence of specific risk alleles for various types of addictions [81].

### 1.7. Different Allelic Frequencies of the Dopamine D3 Receptor and the Opioid Receptor in Opioid Use Disorder

We have completed two related studies utilizing the GARS in a total of 160 diagnosed chronic opioid users, analyzed individually (1) pain patients with OUD, *n* = 121; (2) African-American buprenorphine MAT patients with OUD; *n*= 39; and (3) combined samples from studies 1 and 2, *n* = 160. The complete genetic findings of these studies will be submitted elsewhere. A brief summary of the overall demographics is provided in Table 3, Table 4 and Table 5 of the present report.

#### 1.7.1. Study 1: Opioid Use Disorder Patients in Pain Clinics

Stable, chronic OUD patients (*n* = 121) with pain (overall pain score > 6 out of 10) were recruited from pain clinics in San Antonio and Austin Texas, New York and Idaho. Study protocols were reviewed and approved by the University of Vermont, School of Medicine (Burlington, VT), and PATH Foundation (NY) Institutional Review Boards (IRBs) (registration #IRB00002334) [82]. The de-identified genotyping data conformed to standard HIPAA and Genetic Information Non-Discrimination Act (GINA) practices. The participants provided a written informed consent approved by the respective IRBs. For the entire population, the average morphine milligram equivalent (MME) was 68 mg/d with a range 20–600 mg/day. The MME for males was 102 mg/day, with a range 30–600 mg/d. The MME of Females was 45 mg/d with a range 20–180 mg/d; the duration of treatment in each pain clinic was > 12 months. Subjects’ demographics are displayed in Table 3.

**Figure 2:**
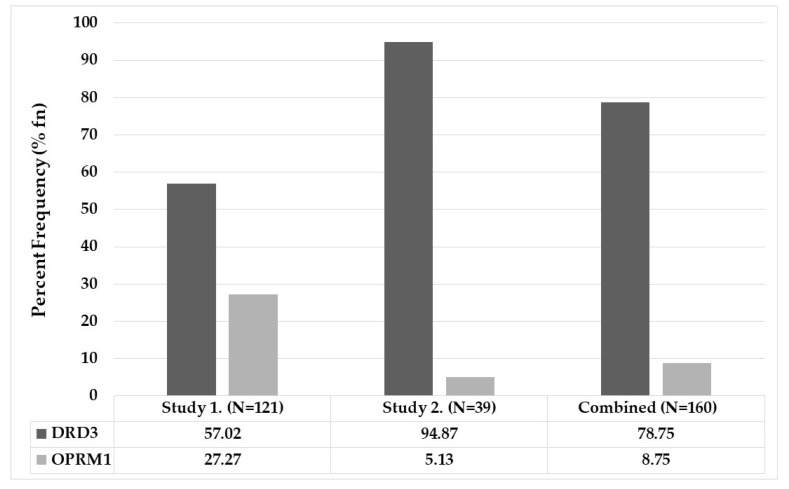
Percent frequencies of DRD3 and OPRM1.

Utilizing the entire GARS panel, the subjects were subsequently genotyped according to previously published methods [81,83]. The frequency and percent of total calls (rank ordered) for the DRD3 (rs6280) risk alleles (Figure 2) were significantly higher (*p* < 0.05) at 57.02% (rank order 7) compared to the OPRM1 (rs1799971) at 27.27% (rank order 10).

#### 1.7.2. Study 2: Opioid Use Disorder Patients at Howard University

This NIH funded study was focused on OUD patients of African ancestry (*n* = 39) receiving buprenorphine MAT. Participants were recruited from both the Howard University Mental Health Center as well as an affiliated community provider office (Medical Home Development Group). Study participants had histories of heroin intake and not opioid medication abuse. Full participation included five study visits at 30-day intervals, questionnaire administration (including RDS Inventory) on visits one and five, and a 30-day supply of a nutraceutical or placebo per visit. Genetic addiction risk profiles were determined utilizing published methods [83], i.e., GARS. Subjects’ demographics are displayed in Table 4. The human subjects research activities described here were approved by the Howard University Internal Review Board (IRB# 17-MED-50).

#### 1.7.3. Combined Analyses of Pain OUD Study #1 and HU- OUD Study #2

In this approach, participants from the Pain Study #1 and the Howard University OUD Study #2 are combined. As expected, the combined group provided the same basic picture, whereby the resultant data independently confirmed that the frequency and percent of total calls (rank ordered) that risk alleles for the DRD3 (rs6280) were significantly higher (*p* <0.05) at 78.75 % (rank order 2) compared to the OPRM1 (rs1799971) at 8.75 % (rank order 11). Subjects’ demographics are displayed in Table 5.

Percent frequency of the DRD3 polymorphism the Pain Clinic study is 57.02; in the HU-OUD study it is 94.87; in the combined study it is 78.75. Percent frequency of the OPRM1 polymorphism the Pain Clinic is 27.27; in the HU-OUD study it is 5.13; in the combined study it is 8.75 (Figure 2). Importantly, the frequency of the OPRM1 risk allele in the predominantly African-American cohort (Table 2, Figure 2) is very low compared to the mostly Caucasian cohort with only 4% African Americans (Table 3, Figure 2), consistent with previous findings 69,71which showed that the risk allele (G) of A118G in rs1799971 is less prevalent in people of African ancestry [54,60,62]. The risk allele (G) of OPRM1 in OUD African Americans in Study 2 was similar to the aggregate allele frequency of 3.1% in the general population as reported by the ALFA Project with dbGaP data [84]. For Caucasians, the dbGaP ‘European’ population frequency was used for comparison, at 13.3% vs. 27.3% in Study 1 OUD patients. Therefore, rs1799971 frequency at least doubled in OUD participants of Study 1.

The DRD3 polymorphisms seems ubiquitous in African-American participants of the HU-OUD Study 2 group at 94.87% vs. 28.35% in African Americans of the ALFA dbSNP database [85], amounting to a 3.34-fold increase in frequency of DRD3 rs6280 in African Americans with OUD. The Pain Clinic–OUD subjects in Study 1 had a frequency of only 57% vs. 67.45% for rs6840 ‘T’ allele in general European populations in the ALFA Project- dbGAP data [86]. Thus, our data show differential frequencies in mostly African American vs. mostly European cohorts with OUD.

## 2. Discussion

Despite extensive research, the neurobiological vulnerability risk factors for OUD have not yet been conclusively determined. The dopaminergic mesocorticolimbic circuitry plays a critical role in OUD as well as in other addictions. Here we report a higher allelic frequency of a polymorphic risk allele of the DRD3 (rs6280) receptor compared to unchanged OPRM1 rs179971 in African American OUD subjects. Caucasians show a higher frequency of the OPRM1(rs1799971) and a slightly lower frequency of the DRD3 risk allele. DRD3 plays a significant role in OUD in Caucasians, but as reported by Abijo et al. several studies show rs2654754, rs9288993, and rs1486009 DRD3 are more prevalent in Caucasians. These SNPs were not evaluated in the current study. In the work of Li et al., [59] NAc DRD3 along with DRD1, DRD2, and DAT are implicated in the pathophysiology of opioid addiction. Moreover, brain-derived neurotrophic factor overexpression in NAc can induce upregulation of DRD3 and DAT, which is helpful to reduce the withdrawal symptoms and craving induced by environmental cues in heroin-addicted rats. In addition, Frances et al. [87], investigating the role of DRD3 in morphine-induced conditioned place preference (CPP), used DRD3 knockout mice (D3-KO). A DRD3-selective partial agonist inhibited the expression of morphine-CPP in wild-type but not D3-KO mice, confirming the role of DRD3 in the expression of conditioned effects of morphine. Recent studies suggest that DRD3 is involved in opioid self-administration, but it remains unclear whether altered DRD3 availability is a risk factor for the development of OUD. To that end, Gardner et al. [49] investigated the role of DRD3 receptor in the different phases of opioid addiction using the D3-KO mice. During acquisition and maintenance of self-administration, D3-KO mice self-administered faster and greater amount of heroin as compared to wild-type mice. D3-KO mice also exhibited greater motivation to self-administer heroin reward under progressive-ratio reinforcement, as well as elevated heroin seeking on extinction and reinstatement procedures. Moreover, deletion of DRD3 resulted in elevated baseline levels of extracellular dopamine in the NAc, leading to higher basal levels of locomotion, and diminished NAc DA and locomotor responses to lower doses of heroin. These findings suggest that DRD3 is critically involved in regulatory processes that limit opioid intake via dopamine-related mechanisms. Deletion of DRD3 also increases opioid-intake and opioid-seeking behaviors. Therefore, decreased DRD3 availability in the brain as found in our genetic studies described herein may be a risk factor for the development of OUD.

Other work from Gardner’s group [88] also addressed the use and abuse of prescription opioid analgesics, particularly oxycodone. Using a rat model, the authors found that pretreatment with novel high affinity DRD3 antagonists/partial agonists, CAB2-015 and BAK4-54 dose-dependently decreased oxycodone self-administration evident in the downward shift of the oxycodone dose-response curve. Moreover, repeated administration of CAB2-015 or BAK4-54 promoted extinction and inhibited oxycodone-induced reinstatement of drug-seeking behavior. The fact that DRD3 antagonist reduced sucrose self-administration that is facilitated by opioids [31,89] as well suggests that DRD3 antagonists (e.g., CAB2-015 or BAK4-54) may be suitable alternatives or adjunctive to opioid-based medications currently used clinically in treating OUD. In summary, the mechanism of how the DRD3 rs6280 SNP, a missense variant, interacts with other gene products to modulate reward mechanisms is not fully known. Thus, an increase in frequency may be inherently different than a decrease in frequency depending on the molecular partners used to respond to stimuli. In our study, only DRD3, and not OPRM1, was significantly changed from the general population as reported by the ALFA- dbGAP. More work is needed to determine the precise impact of expression of the genetic variant. The OPRM1 variant showed double the frequency in Caucasians only.

These and other data along with our finding of changes in the frequency of the DRD3 (rs6280) risk allele relative to the OPRM1 (rs1799971) in African Americans raise the question concerning the possibility that dopaminergic mechanisms play an even more significant role in OUD in Black persons than opioidergic receptor genetic deficit, whereas in White persons, OPRM1 may be more dominant. Indeed, dopaminergic gene polymorphisms underlying aberrant drug behaviors lead to dysregulation in the complex interaction among neurotransmitters (primarily dopaminergic and opioidergic) involved in the BRC, clinically evident as the RDS umbrella of conditions. As a result, individuals with a family history of alcoholism or other addictions may be born as being “several drinks behind” [90] the rest of the world due to a deficiency in the ability to produce or use these neurotransmitters. Alternatively, chronic exposure to stress [91], alcohol or other substances also can lead to a similar corruption of the BRC function [21]. Accordingly, Blum’s group [92] evaluated the potential association of four variants of dopaminergic candidate genes in RDS -DRD1, DRD2, DAT1 and dopamine beta-hydroxylase- across five generations. Their results support the putative role of dopaminergic polymorphisms in RDS behaviors. Furthermore, as it relates to the present paper, the earlier published by Blum et al. [92] study shows the importance of a nonspecific RDS phenotype and informs an understanding of how evaluating just a single subset RDS behaviors may lead to spurious results. Utilization of a nonspecific “reward” phenotype may be a paradigm shift in future association and linkage studies involving dopaminergic polymorphisms and other neurotransmitter gene candidates.

Ultimately, both dopaminergic and opioidergic processes are involved in OUD. However, we are poised to suggest that because the brain is not carved out as displayed by the DSM-5 psychiatric guidelines, at least in our studied cohort, the DRD3 risk allele seems to be over-represented in African American subjects and under-represented in Caucasian OUD subjects. Our novel findings are suggestive of a prominent role of dopamine type receptors in specific ethnicities in our cohort. While these data must be met with caution, novel targets directed at overcoming opioid seeking may reside in induction of “dopamine homeostasis” rather than specific opioidergic targets *per se*. In fact, Volkow, in a recent conversation with Czerin [93], suggested that DRD3 represented a new and novel therapeutic target to assist in overcoming the American Opioid Crisis.

An increased prevalence of a particular SNP or variant in African Americans does not always translate to similar addictive behaviors across different ethnic groups. Specifically, there is a differential impact of gene variants on opioid metabolism in Europeans vs. African Americans. As an example, -2044C/A haplotype at the Mu opioid 1 receptor modulates the reward pathway to have a net negative effect on OUD, whereas European Americans with this allele are more susceptible to OUD than African Americans [71]. By contrast, a higher frequency of the *1B allele in the P450 system is present in African Americans, and this allele confers the extended metabolizer phenotype [94]. Moreover, the possibility of increasing chances of relapse in African Americans undergoing opioid replacement therapy with Buprenorphine/Suboxone, carriers of the CYP3A4, Cytochrome P450 3A4 showing higher relapse has been reported by Chapman’s group [94]. Hence, our findings require further attention and validation in diverse ethnic groups.

## 3. Summary

In keeping with the notion that common neurogenetic mechanisms underlie impulsive, compulsive and addictive disorders, we propose that DRD3 may be a major modulator of reward, and polymorphisms in DRD3 significantly contribute to reward deficiency and a spectrum of disorders. Our studies here make the case that treatment of RDS requires consideration of DRD3 as a therapeutic target. Furthermore, an understanding of the mechanistic impact of DRD3 downstream signaling is necessary. These studies additionally support the notion that some reward genes and their variations are differentially expressed in people of different ancestry. We investigated OPRM1 and DRD3, as examples, but consideration of ancestry-dependent disparate gene/variant expression must be foremost in this era of precision medicine for inclusion of all people. All in all, it may be useful to segregate the multidimensional construct of addiction based on the underlying genetic underpinning in accordance with the Research Domain Criteria. This may provide a sound foundation for understanding potential interactions among clinical manifestations, (epi)genetic factors and therapeutic targets that are linked to the root cause of brain disorders such as OUD. Such an approach would be consistent with the former director of the NIMH Steven Hyman’s call for a new genetic map to display psychiatric phenotypes [95].

## 4. Conclusions

While opioidergic mechanisms are certainly involved in OUD, dopamine-related receptors may have more primary influence on opioid-seeking behavior. Taken together, these findings offer the possibility that novel and improved neuropharmacological therapeutic approaches to OUD may focus on regulation of dopaminergic homeostasis via the DRD3 pathway. We encourage more in-depth research on this important topic [49,84,87,88,92,96,97,98,99,100,101,102,103,104,105,106,107,108,109,110,111,112].

## Figures and Tables

**Table 1:** DRD3 gene polymorphisms and RDS—Not specific for Opioids (a sampling).

Gene	Polymorphism	Study Findings	Reference
*DRD3* OMIM 126451	rs6280	Significant interaction for BDNF Val66Met Val/Val genotype with, both DRD3 Ser9Gly Ser/Ser and Ser/Gly SNPs in bipolar-II patients (*p* = 0.027 and 0.006, respectively).	Lee et al. [57,58]
	DRD3 KO mice	DRD3 knockout mice (DRD3 KO): hypoalgesia, lower morphine-induced tolerance and attenuated withdrawal signs compared with the wild type mice.	Li et al. [59]
	rs6280	Upregulation of DRD3 in the striatum of alcohol preferring (P) and high alcohol drinking (HAD)rats through DNA microarrarys, confirmed by qRT-polymerase chain reaction.	Vengeliene et al. [60]
	rs6280	Respectively decreased and increased parietal and frontal P300 amplitudes in Gly9 homozygotes versus Ser9 carriers.	Mulert et al. [61]
	BalI	Impulsive alcohol dependent patients were more frequently heterozygous for DRD3 BalI in comparison to both, alcohol-dependent patients with lower impulsivity ratings (OR = 2.51, *p* < 0.02) and to healthy controls (OR = 2.32, *p* < 0.03).	Limosin et al. [62]
		High sensation-seeking score was more frequent in homozygous for both alleles than those with a low sensation-seeking score under 24 (*p* < 0.04) or controls (*p* = 0.03).	Duaux et al. [63]
		Binging on a sucrose solution increased the expression of DRD3 gene (NAc > caudate, putamen) and decreases that of the DRD2 and of the preproenkephalin and preprotachykinin genes.	Spangler et al. [64]
	MscI/BalI	Increased homozygosity in cocaine dependence (29.8%) vs. controls (46.9%) particularly in those with chronic cocaine consumption for > 10 years (25%) and > 15 years (46.5%).	Comings et al. [65]

**Table 2:** OPRM1 gene polymorphisms and RDS—Not specific for Opioids (a sampling).

Gene	Polymorphism (Study Conditions)	Study Findings	Reference
*OPRM1* OMIM 610064	rs1799971	Independent of session, smokers homozygous for the wild-type OPRM1 A allele exhibited significantly higher levels of non-dominant mu opioid receptor A118G than smokers carrying the G allele in bilateral amygdala, left thalamus, and left anterior cingulate cortex.	Ray et al. [77]
	rs1799971	Found a significant association for both A118G and C1031G polymorphisms and opioid dependence. The G allele is more common in the heroin-dependent group (39.5% and 30.8% for A118G and C1031G polymorphisms, respectively) when compared to the controls (29.4% and 21.1% for A118G and C1031G polymorphisms, respectively). *this is the only study reporting C1031G	Szeto et al. [73]
	rs1799971	There was a significant overall association between genotypes with an 118G allele and alcohol dependence (*p* = 0.0074). The attributable risk for alcohol dependence in subjects with an 118G allele was 11.1%	Bart et al. [78]
	OPRM1 KO mice	Wild type mice consumed more alcohol than heterozygous or homozygous MOR KO mice (female KO mice > male KO mice). MOR KO mice exhibited less ethanol reward in a conditioned place preference paradigm (females < males).	Hall et al. [79]

**Table 3:** Subject demographics for Study 1.

Population		All	Male	Female
Number (*n*)		121	55 (45%)	66 (55%)
Average Age (*n* = 121)		53	54	53
Ethnicity				
	Caucasian	67%		
	Hispanic	17%		
	Unknown	10%		
	Black or African American	4%		
	Asian	2%		

**Table 4:** Subject demographics, gender, age and ethnicity for study 2.

Population		All	Male	Female
Number (*n*)		39	28 (72%)	11(28%)
Ethnicity				
	Black or African American	97%		
	Unknown	3%		

**Table 5:** Combined subject demographics, gender, age and ethnicity.

Population		All	Male	Female
Number (*n*)		160	83 (52%)	76(48%)
Average Age (*n* = 121)		53	54	53
Ethnicity				
	Caucasian	51%		
	Black or African American	27%		
	Hispanic	13%		
	Unknown	8%		
	Asian	1%		

## Data Availability

Not applicable.
